# Targeting breathlessness in heart failure

**DOI:** 10.1002/ehf2.12556

**Published:** 2020-01-08

**Authors:** Andrew J. Stewart Coats

**Affiliations:** ^1^ San Raffaele Pisana Scientific Institute Rome Italy

This article refers to ‘Oral modified release morphine for breathlessness in chronic heart failure: a randomized placebo‐controlled trial’, by Johnson MJ *et al*., published in this issue on pages 1149–1160.

In this issue, we see reported a double‐blind, randomized controlled trial (RCT) of 20 mg daily oral modified release morphine in 45 patients with HFrEF with the aim to reduce the severity of breathlessness (dyspnoea).^1^ The idea is not novel. Several trials going back to 1990s have shown that morphine can relieve exertional or situational dyspnoea in heart failure patients.^2^, ^3^, ^4^ These have built on a larger literature on COPD, cancer, and myocardial infarction (MI)‐induced dyspnoea as well as in palliative care, often for both pain and dyspnoea relief.^5^

The trial did not achieve its primary aim that of a significant reduction in the severity of breathlessness at week 4. This is not surprising, for the trial was underpowered due to poor recruitment, with only 45 of planned 346 patients being recruited. This does not mean the idea was incorrect, and the authors are to be congratulated for seeking to explore this interesting therapeutic idea and worthy aim in a proper RCT, when so few are conducted in HF with the aim to relieve disabling symptoms, rather than the more common aim to improve mortality and hospitalization rates. No serious adverse effects were seen. From such a neutral trial, it is impossible to say anything definitely concerning secondary end points, especially when it closed early, so seriously underpowered. What can be said, however, is that the trial never had a chance, especially when combining the poor recruitment with poor compliance with the medication. The proportion of tablets taken were below one‐half expected (39–51%) in the active therapy group, perhaps due to side effects (nausea and constipation are obvious candidates); perhaps other reasons.

Does this result mean the idea should be forgotten; definitely not. There are far too few treatments that improve symptoms on HF and that is a result of far too few trials looking at treatments with that treatment objective in mind. Perhaps we could improve on the design. We usually simplify a symptom. We talk of dyspnoea as being present or absent, but resting dyspnoea is not the most common report; more common is exertional or nocturnal dyspnoea. Perhaps a patient doesn't want to take a tablet to reduce dyspnoea when they are not getting the symptom. Perhaps we should look at a short‐acting opiates, taken akin to a nitrate spray by an angina patient, to enable a physical activity to be competed more easily such as climbing stairs or sexual intercourse, rather than always assuming all drugs need to be present for 24 hours. Tolerance may be better, and end points more focussed on the precise mode of action of the drug may have a better chance of being reached. In this regard, a much earlier trial showed significant efficacy of the mild opiate dihydrocodeine in reducing the sensation of dyspnoea during a progressive exercise test and being associated with a reduction in ventilatory drive, and leading to a significant increase in exercise tolerance,^6^ perhaps because it was given specifically timed to the performance of a bout of exercise.

Another possibility is that some of the patients have central sleep apnoea^7^, ^8^ where the pathophysiological mechanisms of nocturnal dyspnoea are partly related to chemoreflex hypersensitivity and periodic episodes of hyperventilation.^9^, ^10^ Similar episodes can occur during the day.^11^ Morphine can suppress these and may relieve nocturnal central sleep apnoea and thereby even improve daytime sleepiness despite oral morphine being taken. We have not seen the last of a potential role for opiates in HF, and we require more of these innovative, physiologically focussed trials. Suppressive, the exaggerated drive to ventilation in HF and the associated dyspnoea is a worthy treatment aim for future clinical trials.

## Conflict of interest

Nothing related to this work. Outside of this work, in the last 3 years, Professor Coats declares having received honoraria and/or lecture fees from AstraZeneca, Menarini, Novartis, Nutricia, Respicardia, Servier, Stealth Peptides, Vifor, Actimed, Enopace, Faraday, and Gore.
